# Modelling bronchial epithelial-fibroblast cross-talk in idiopathic pulmonary fibrosis (IPF) using a human-derived in vitro air liquid interface (ALI) culture

**DOI:** 10.1038/s41598-023-50618-y

**Published:** 2024-01-02

**Authors:** Sarah L. Barron, Owen Wyatt, Andy O’Connor, David Mansfield, E. Suzanne Cohen, Tomasz M. Witkos, Sam Strickson, Róisín M. Owens

**Affiliations:** 1 Chemical Engineering and Biotechnology Department, University of Cambridge, Cambridge, UK; 2 Research and Early Development, Respiratory and Immunology, Bioscience Asthma and Skin Immunity, AstraZeneca, Cambridge, UK; 3 Imaging and Data Analytics, Clinical Pharmacology and Safety Sciences, AstraZeneca, Cambridge, UK; 4 Analytical Sciences, Bioassay, Biosafety and Impurities, BioPharmaceutical Development, AstraZeneca, Cambridge, UK

**Keywords:** Biological models, Gene expression analysis

## Abstract

Idiopathic Pulmonary Fibrosis (IPF) is a devastating form of respiratory disease with a life expectancy of 3–4 years. Inflammation, epithelial injury and myofibroblast proliferation have been implicated in disease initiation and, recently, epithelial-fibroblastic crosstalk has been identified as a central driver. However, the ability to interrogate this crosstalk is limited due to the absence of in vitro models that mimic physiological conditions. To investigate IPF dysregulated cross-talk, primary normal human bronchial epithelial (NHBE) cells and primary normal human lung fibroblasts (NHLF) or diseased human lung fibroblasts (DHLF) from IPF patients, were co-cultured in direct contact at the air–liquid interface (ALI). Intercellular crosstalk was assessed by comparing cellular phenotypes of co-cultures to respective monocultures, through optical, biomolecular and electrical methods. A co-culture-dependent decrease in epithelium thickness, basal cell mRNA (P63, KRT5) and an increase in transepithelial electrical resistance (TEER) was observed. This effect was significantly enhanced in DHLF co-cultures and lead to the induction of epithelial to mesenchymal transition (EMT) and increased mRNA expression of TGFβ-2, ZO-1 and DN12. When stimulated with exogenous TGFβ, NHBE and NHLF monocultures showed a significant upregulation of EMT (COL1A1, FN1, VIM, ASMA) and senescence (P21) markers, respectively. In contrast, direct NHLF/NHBE co-culture indicated a protective role of epithelial-fibroblastic cross-talk against TGFβ-induced EMT, fibroblast-to-myofibroblast transition (FMT) and inflammatory cytokine release (IL-6, IL-8, IL-13, IL-1β, TNF-α). DHLF co-cultures showed no significant phenotypic transition upon stimulation, likely due to the constitutively high expression of TGFβ isoforms prior to any exogenous stimulation. The model developed provides an alternative method to generate IPF-related bronchial epithelial phenotypes in vitro, through the direct co-culture of human lung fibroblasts with NHBEs. These findings highlight the importance of fibroblast TGFβ signaling in EMT but that monocultures give rise to differential responses compared to co-cultures, when exposed to this pro-inflammatory stimulus. This holds implications for any translation conclusions drawn from monoculture studies and is an important step in development of more biomimetic models of IPF. In summary, we believe this in vitro system to study fibroblast-epithelial crosstalk, within the context of IPF, provides a platform which will aid in the identification and validation of novel targets.

## Introduction

Idiopathic Pulmonary Fibrosis (IPF) is a severe and aggressive form of pulmonary fibrosis that results in the accumulation of fibrotic scar tissue and decreased respiratory function including inefficient gas exchange and difficulty breathing^1^. Known risk factors include smoking cigarettes, lung injury and autoimmune conditions, however, the exact etiology remains unclear^1,2^. Since 2014 only two medicines have been approved by regulators (Pirfenidone and Nintedanib)^3^, however, these therapies have limited efficacy and a high burden of side-effects. Consequently, lung transplantation is the only curative treatment, which is not without risk, and the prognosis of death from IPF remains at 3–4 years. One reason for the high drug attrition rates associated with translational research is associated with the use of animal models, which fail to recapitulate human physiology and anatomy. Additionally, the most commonly used in vivo models of IPF, chemically induce fibrosis with Bleomycin or Fluorescein isothiocyanate (FITC) which fail to recapitulate the chronic and progressive nature of human IPF^3^. Thus, there is a need for biomimetic in vitro drug screen platforms which recapitulate (disease) physiology and allow for the interrogation of novel pathways and identification of novel drug targets.

**Figure 1:**
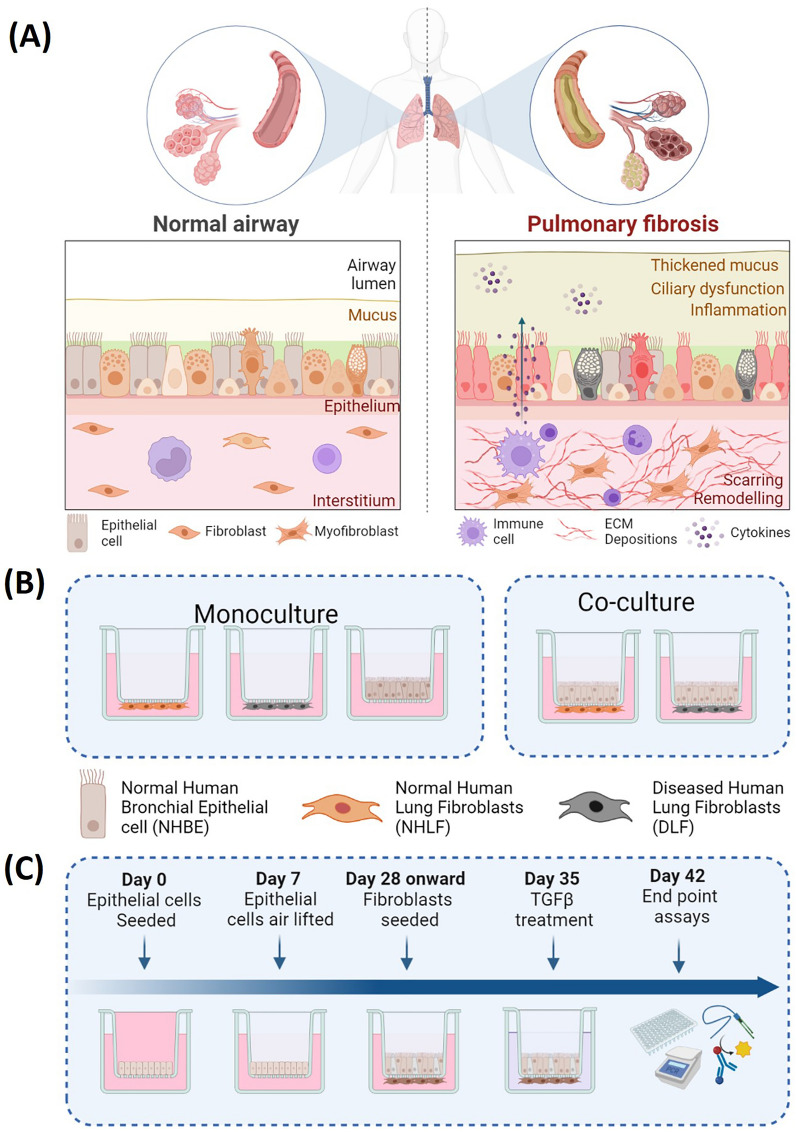
Schematic representation of the human respiratory environment in health and fibrotic disease (**A**). Experimental setup of in vitro cell cultures showing the various configurations (**B**). Primary human fibroblasts (healthy or diseased) and epithelial cells were grown in monoculture (left) or co-culture (right). Optimised experimental protocol (**C**) where epithelial cells were seeded (day 0), air-lifted (day 7) and differentiated (days 7–28) before fibroblasts were added to the underside of the membrane inserts (day 28). After one week of fibroblast culture (either in mono- or co-culture), cell groups were stimulated with TGFβ-1 for a further week before end point assays were performed. NHBE = Normal Human Bronchial Epithelial cell; NHLF = Normal Human Lung Fibroblast; DHLF = Diseased Human Lung Fibroblasts. Schematic created with Biorender.com.

The role of fibroblast-to-myofibroblast transition (FMT)^4–6^ and epithelial-to-mesenchymal transition (EMT)^7–9^ in IPF pathophysiology has been widely characterized in two-dimensional (2D) in vitro monocultures. However, contemporary views now appreciate the role of the ECM, dysfunctional muco-ciliary clearance and epithelial-fibroblast cross-talk in disease progression (Fig. 1A)^2,10^. Despite this, many in vitro IPF models still lack the three-dimensional (3D), multi-cellular, environment of the native lung tissue, which is known to enhance differentiation and response to drug challenges^11–14^. Furthermore, evidence points to the pathological role of epithelial barrier integrity in inflammatory lung disease^10,15–19^. For example, primary in vitro models of asthmatic^20,21^ and Chronic Obstructive Pulmonary Disorder (COPD)^22,23^ epithelium display altered tight junction protein expression and barrier permeability. However, few studies have looked to characterize the pathogenic implications of epithelial barrier (dys)function in the progression of IPF.

In order to investigate the role of fibroblasts in regulating epithelial barrier integrity and epithelial-mesenchymal-transition (EMT), normal human bronchial epithelial (NHBE) cells were co-cultured with either normal human lung fibroblasts (NHLF) or diseased (IPF-derived) human lung fibroblasts (DHLF). Subsequently, the role of epithelial-fibroblast crosstalk in IPF initiation was assessed by exposing co-cultures to transforming growth factor beta (TGFβ-1), a potent driver of fibrosis^24–26^. In contrast to routine monoculture studies, commonly used in drug discovery settings, here, the effect of direct epithelial-fibroblast co-culture on barrier integrity, gene expression and cytokine release was characterized. Our results show that epithelial and fibroblastic populations give differential responses when grown in monoculture vs co-culture and when exposed to a pro-inflammatory environment. This holds implications for any translation conclusions that may be drawn from monoculture studies and may provide a more relevant model for future drug studies or identification of new therapeutic targets.

## Methods

### Cell culture

Normal Human Bronchial Epithelial cells (NHBEs) from non-smokers (CC-2540; Lonza, Switzerland; ID 88372; ID 90330) were initiated into EpiX™ basal media (STEMCELL Technologies, USA). Once confluent, cells were washed with Hanks Balanced Salt Solution (HBSS, without Ca^2+^; ThermoFisher, USA), harvested using TrypLE express (ThermoFisher, USA) and neutralised with HBBS (with Ca^2+^). NHBEs were seeded onto 0.4 µm pore, polyester 24 well inserts Transwell ® inserts (Corning ® Costar ®, USA) at a density of 3.5 × 10^5^ cm^2^. Prior to seeding, all Transwells ® were coated with CellAdhere™ type 1 human collagen (STEMCELL Technologies, USA) overnight. Cells were cultured for 7 days until confluent before apical media was aspirated (air-lifted) and basolateral media switched to PneumaCult™ ALI differentiation media (STEMCELL Technologies, USA) supplemented with 0.2% heparin (STEMCELL Technologies, USA), 0.5% hydrocortisone (STEMCELL Technologies, USA) and 0.2% Pen/strep (ThermoFisher, USA).

Human Lung Fibroblasts (NHLF; From healthy non-smokers; ID 85620; ID 90350; ID 92283) or Diseased Lung Fibroblasts (DHLF; From IPF patients, ID 85609; ID 95650; ID 95554) were initiated into FGM™-2 fibroblasts growth medium-2 BulletKit™ (Lonza, Switzerland). Once confluent, cells were washed with PBS × 1, harvested with TrypLE express (ThermoFisher, USA) and neutralized with neutralizing solution (HBSS containing 5% FBS; ThermoFisher, USA). Cells were seeded at a density of 3 × 10^4^ cm^2^ on the underside of a Transwell ® insert as a monoculture or as a co-culture (NHBEs on apical surface of insert). Once Co-culture was initiated, media was changed in all wells to co-culture media, which contained an 80:20 media ratio (NHBE media: fibroblast media). This ratio was chosen based on cell proliferation (Incucyte ®; Sartorius, Germany) and area under the curve (AUC) analysis (Figure S1). Media was replaced every 2–3 days and all cultures were grown for a further 7 days before treatment. For data presented in this work n = 2 donors for NHBEs, n = 3 donors for NHLFs and n = 3 donors for DHLFs. Data presented were conducted over 2–3 experimental repeats, with the overall n number for each condition given in individual captions.

### TGFβ treatment

To induce a proinflammatory environment, cell cultures were treated with recombinant human TGFβ-1 protein (240-B; R&D Systems, USA) at a concentration of 1 ng/ml, in the basal compartment of cultures, for 7 days. Media with fresh TGFβ-1 was replaced every 2–3 days.

### TEER

TEER measurements were taken using the EVOM 3 and STX-2 plus electrode (World Precision Instrument, USA). Prior to recordings with cells at ALI, media was replaced on apical and basal sides of the Transwell ® insert and allowed to equilibrate for 30 min. Cells were removed from the incubator and allowed to reach room temperature. TEER values are reported as Ω cm^2^ where *TEER* = (Raw value – blank, no cell, value) * 0.33 cm^2^ (growth surface area of insert) and averaged as 3 readings per well. Values are presented as the mean ± SD for each cell group (n = 8–32).

### Cell lysis, RNA extraction and cDNA synthesis

After treatment and final TEER measurements, Transwell ® inserts containing either monocultures or co-cultures were placed into a tube containing lysis buffer (Beckman Coulter©, Switzerland) and incubated at 37 °C for 25 min. RNA was extracted through multiple wash and magnetic bead binding steps, using the RNAdvance tissue kit (Beckman Coulter ©**,** Switzerland). RNA samples were eluted in 20 µl dH_2_O, added to reverse transcriptase mix (High-capacity RNA-to-cDNA™ kit; ThermoFisher; USA), sealed and briefly centrifuged to remove any bubbles. Samples were transferred to a 96 well thermal cycler (Eppendorf Mastercycler ® personal, Germany) and incubated at 37 °C for 60 min. The reaction was stopped by heating to 95 °C for 5 min and held at 4 °C. cDNA samples were diluted 1:3 in dH_2_O and stored at − 80 °C until needed.

### Real time q-PCR

cDNA samples were thawed and transferred to a 384 well q-PCR plate (MicroAmp™ EnduraPlate™; ThermoFisher, USA). TaqMan™ probes (ThermoFisher; USA) were multiplexed with housekeeper gene and gene of interest (Table 1). Reaction mix was prepared by adding MasterMix (TaqMan™; ThermoFisher, USA), housekeeping probe and gene of interest probe to each cDNA sample using the ECHO525 acoustic handler (Beckman Coulter ©**,** Switzerland). Plates were briefly centrifuged to remove any bubbles then run on a Thermo QuantStudio™ 7 Flex real-time PCR system (40 cycles). Data for real time q-PCR are presented as fold change (Log(2)) from control group. The 2-(ΔΔCt) method was used for data analysis, where cycle threshold (CT) represents the PCR cycle number where the fluorescence single becomes distinguishable from background noise where:

$$\Delta {\text{C}} = {\text{Ct}}\left( {\text{gene of interest}} \right) \, {-}{\text{ Ct}}\left( {{\text{GAPDH}}} \right){\text{ and }}\Delta \Delta {\text{C }} = \, \Delta {\text{C}}\left( {\text{treated sample}} \right) - \, \Delta {\text{C}}\left( {\text{untreated sample}} \right).$$

**Table 1:** List of TaqMan™ primers and probes used for real time q-PCR.

Gene	Marker	Catalogue no.	Dye	Size (rxns)
*GAPDH*	House keeping	Hs02786624_g1	VIC-MGB	250
*P63*	Basal	Hs00978340_m1	FAM-MGB	250
*KRT5*	Basal	Hs00361185_m1	FAM-MGB	250
*SCGB1A1* (CC16)	Secretory/club cell	Hs00171092_m1	FAM-MGB	250
*DNI2*	Ciliated	Hs00224913_m1	FAM-MGB	250
*FOXJ1*	Ciliated	Hs00230964_m1	FAM-MGB	250
*MUC5AC*	Secretory/goblet	Hs01365616_m1	FAM-MGB	250
*MUC5B*	Secretory/goblet	Hs00861595_m1	FAM-MGB	250
*CDH1 (E-CAD)*	Epithelial Cadherin	Hs01023894_m1	FAM-MGB	250
*TJP (ZO-1)*	Tight junction	Hs01551871_m1	FAM-MGB	250
*FN1*	ECM protein/Fibronectin	Hs01549976_m1	FAM-MGB	250
*COL1A1*	ECM protein/Collagen	Hs00164004_m1	FAM-MGB	250
*VIM*	Vimentin	Hs00958111_m1	FAM-MGB	250
*ACTA1 (ASMA)*	Smooth muscle actin	Hs00559403_m1	FAM-MGB	250
*CDK1 (P21)*	Senescence	Hs00938777_m1	FAM-MGB	250
*TGFB1*	Transforming growth factor β1	Hs00998133_m1	FAM-MGB	250
*TGFB2*	Transforming growth factor β2	Hs00234244_m1	FAM-MGB	250
*TGFB3*	Transforming growth factor β3	Hs01086000_m1	FAM-MGB	250

The ΔΔCT values were then log transformed to give the relative fold gene expression to untreated controls. Values are presented as the mean ± SD for each cell group (n = 6–11).

### ELISA

After treatment, and prior to final TEER measurements, the supernatant from the basal side of cultures were aspirated and frozen at – 80 °C until needed. Samples were then washed and prepared using the MULTI_SPOT assay system proinflammatory panel 1 human kit (MSD ®, USA) which contains detection antibodies for the inflammatory analytes listed below (Table 2). Samples were read using plate reader (MESO SECTOR S 600; MSD ®, USA) and analyte concentrations (pg/ml) calculated by fitting signals to a calibration curve using DISOVERY WORKBENCH ® version 4.0 (MSD ®, USA). Data is presented as pg/ml or as fold change compared to untreated controls. Values are presented as the mean ± SD for each cell group (n = 4–8).

**Table 2:** List of analytes and typical detection limits used for ELISA.

Analyte	LLOD	LLOQ	ULOQ	Dynamic range	Unit
IFN-γ	0.37	1.76	938	0.37–938	pg/mL
IL-1β	0.05	0.646	375	0.05–375	pg/mL
IL-2	0.09	0.89	938	0.09–938	pg/mL
IL-4	0.02	0.218	158	0.02–158	pg/mL
IL-6	0.06	0.633	488	0.06–488	pg/mL
IL-8	0.07	0.591	375	0.07–375	pg/mL
IL-10	0.04	0.298	233	0.04–233	pg/mL
IL-12	0.11	1.22	315	0.11–315	pg/mL
IL-13	0.24	4.21	353	0.24–353	pg/mL
TNF-α	0.04	0.69	248	0.04–248	pg/mL

*Taken from MSD ® MULTI_SPOT assay system proinflammatory panel 1 human kit manual.

### Histology and immunofluorescence staining

ALI epithelial cultures were fixed in 10% formalin for 24 h and embedded in paraffin. Paraffin sections (4 μm) were mounted on positively charged slides and stained on the Leica BOND Rx platform. For H&E stains, slides were counterstained with Hematoxylin II for 8 min (Roche, Switzerland) and bluing reagent for 4 min (Roche, Switzerland), rinsed with dishwashing detergent, dehydrated with a graded series of ethanol and xylene, and mounted with permanent mounting media. For immunofluorescent staining, antigen retrieval was performed with BOND Epitope Retrieval Solution 1 (Leica, AR9961) for 20 min and endogenous peroxidase was blocked with Novocastra Peroxidase Block (Leica, RE7101) for 20 min. Anti-MUC5AC (ThermoFisher, USA; MA1-21907; 0.0015 μg/ml), anti-MUC5B (Abcam, UK; ab87376; 0.025 μg/ml) and anti-E-cadherin (4A2) (Cell Signaling Technology, USA; 14472; 0.25 μg/ml) diluted in BOND primary antibody diluent (Leica, AR9352) were sequentially added to the samples, with an additional antibody denaturation step performed between each addition, with BOND Epitope Retrieval Solution 1 (Leica, AR9961) for 20 min at 95°C. Anti-MUC5AC was incubated for 60 min, anti-MUC5B for 30 min and anti-E-cadherin for 30 min. Samples were visualized with a two-step polymer detection system: Polink-2 HRP polymer enhancer (10 min) and Polink-2 HRP polymer detector (10 min) (GBI, D41-110). This was followed by incubation with Opal (Akoya Biosciences, FP1496A) diluted in Plus Amplification buffer (Akoya Biosciences, FP1498) for 10 min. Opal was diluted 1:200 for Anti-MUC5AC, 1:150 for anti-MUC5B and 1:50 for anti-E-cadherin.The slides were then stained with DAPI (Akoya Biosciences, FP1490) diluted in BOND wash solution (Leica, AR9590) for 10 min at room temperature. Slides were then rinsed with deionized water and mounted with ProLong Diamond antifade mounting media (Thermo, P36965) before being scanned with Akoya Vectra Polaris.

### Surface contrast and cell morphology analysis using ImageJ

For surface contrast quantification, images of the apical surface of cell cultures were captured using brightfield microscopy (EVOS M7000; ThermoFisher, USA), at the center of each well. Magnification (× 10) and brightness of the lens was kept consistent throughout. Images were converted to 32-bit greyscale and analyzed in ImageJ-Win64 using the auto-threshold, analyze and measure functions to give a histogram plot of grey scale value distribution. In this context, surface contrast is given as the mean grey value, as a percentage of the image area. For each cell condition 3–4 images were averaged and presented as the mean ± SD. For cell/nucleus morphology analysis of H&E stained histology slices, images were converted to 8-bit greyscale, thresholded and analyzed using the particle plugin function. Nucleus size is defined as area in µm^2^. Nucleus roundness is defined as how closely the shape fits a circle, where a value of 1 is a perfect circle, and is calculated as the ratio of the nucleus area by the square of its parameter. Nucleus aspect ratio is defined as the ratio between the major and minor axis of the nucleus shape, where 1 is a perfect circle and infinity is completely linear. For histology 1–2 images were used and nucleus number per image was n = 75–162. For epithelial height, 50 lines per image were drawn (from the apical to basal section of the epithelium), measured and the average taken. Values were presented as the mean ± SD.

### Statistical analysis

Statistical analysis was performed in GraphPad Prism (version 8.0). For pre-treatment conditions an ordinary one-way ANOVA with Tukey’s multiple comparisons test was performed. For post-treatment conditions, comparing untreated to treated, multiple T tests per row with Holm-Sidak multiple comparison test was used. Data are presented as the mean ± SD where each condition contains 2–3 donors, 2–4 biological replicates and 4–8 experimental replicates.

## Results

### Development of the in vitro ALI co-culture model

To generate the model, the two main functional cell types of the respiratory airways, fibroblasts and epithelial cells, were chosen. Primary NHLFs, DHLFs or NHBEs were purchased from commercial sources and grown in either a monoculture or co-culture configuration (Fig. 1B). Although the use of IPF-derived epithelial cells would better reflect the diseased environment, and would provide further insights into IPF dysregulated cross-talk, they are extremely difficult to access. Thus, this model provides an alternative method to generate IPF-related epithelial phenotypes in vitro with mixed donors. For co-cultures, NHBEs were grown on the apical side of a collagen coated Transwell ® insert at the Air Liquid Interface (ALI), with fibroblasts grown on the underside. In this way, the in vivo 3D tissue structure of the upper airway epithelium was mimicked. Following optimization of a media recipe for co-cultures (further details in methods and in Additional file 1, Figure S1), a five week protocol was established (Fig. 1C); NHBEs were cultured in ALI conditions (epithelial monocultures and co-cultures) for three weeks before fibroblasts were seeded on the underside of the Transwell insert (fibroblast monocultures and co-cultures) and treatments began.

### Characterization of cellular morphology

**Figure 2:**
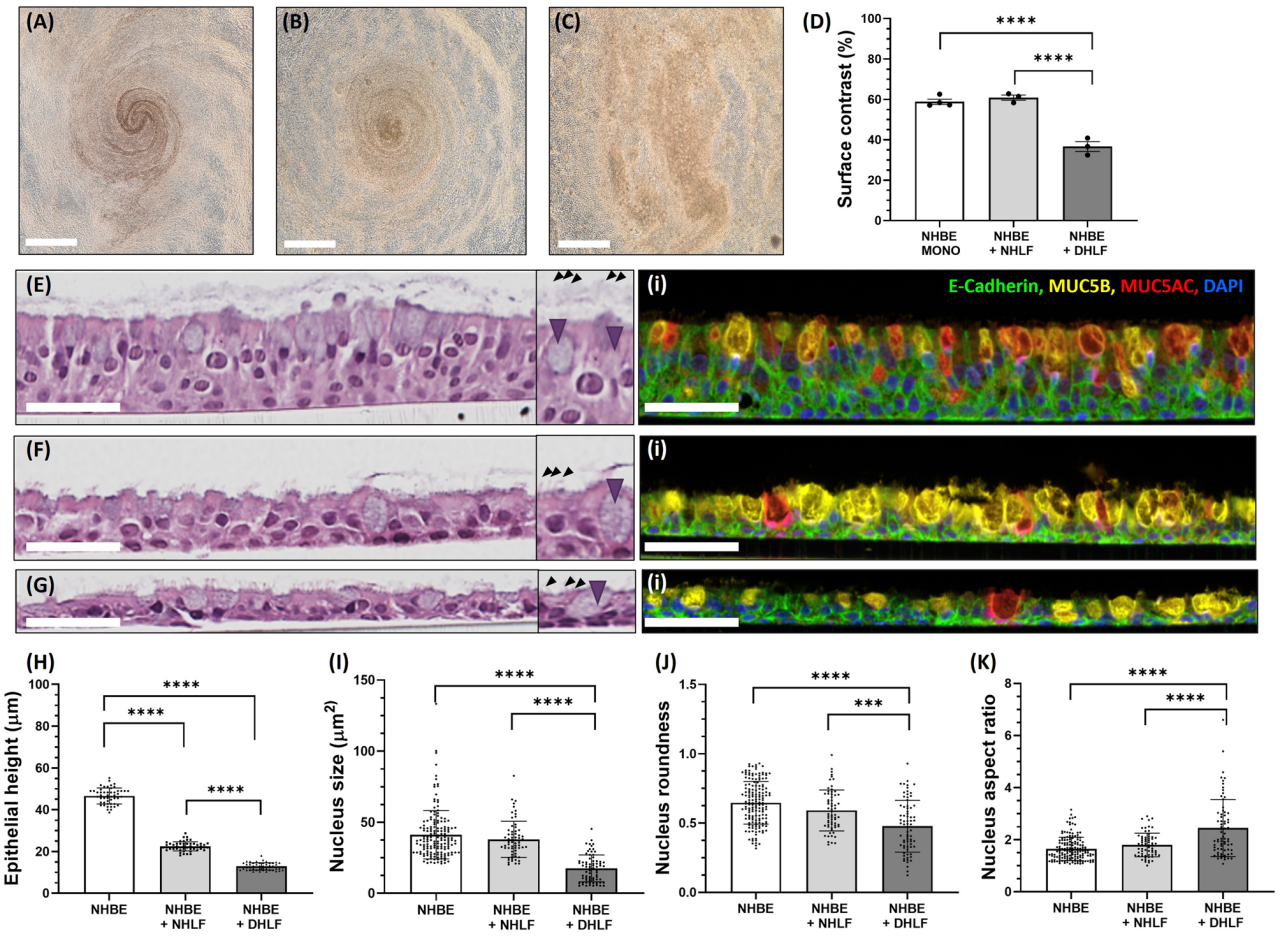
Optical characterisation of in vitro cultures. Representative brightfield images of the apical surface of epithelial monocultures (**A**), healthy fibroblast co-cultures (**B**), and diseased fibroblast co-cultures (**C**). Quantification of apical surface contrast (**D**), as a proxy for mucus coverage, for epithelial monocultures and co-cultures. Scale bar = 1 mm, N = 3 representative images for each condition. Haematoxylin and eosin stain (left) and immunofluorescent stain (right) of representative histological slices of epithelial monocultures (**E**), healthy fibroblast co-cultures (**F**), and diseased fibroblast co-cultures (**G**). Scale bars = 50 µm, insets =  × 1.5 magnification. Purple arrow heads indicate goblet cells and black arrow heads indicate cilia projections. Anti-E-cadherin can be visualised in green (Cell Signaling Technology, 0.25 μg/ml), anti-MUC5B in yellow (Abcam, 0.025 μg/ml), anti-MUC5AC in red (ThermoFisher, 0.0015 μg/ml) and DAPI in blue (Akoya Biosciences). Quantification of cell morphology for representative slices including epithelial height (**H**), nucleus size (**I**), roundness (**J**), and aspect ratio (**K**). Statistical analysis was performed using a one-way ANOVA with Tukey’s multiple comparisons (****, P =  < 0.0001; *** = 0.0001), N = 160 for NHBEs, N = 65 for NHBE + NHLF and N = 75 for NHBE + DHLF. For epithelial height N = 50. Data are presented as the mean ± SD. NHBE = Normal Human Bronchial Epithelial cell; NHLF = Normal Human Lung Fibroblast; DHLF = Diseased Human Lung Fibroblasts.

Respiratory epithelial cells, when cultured at ALI in vitro, differentiate and give rise to multiple cell types found in vivo^27^*.* This includes basal stem cells, mucus-secreting goblet cells and ciliated cells*.* The synergistic functions of mucus and cilia aid in the capture and clearance of noxious stimuli, known as muco-ciliary clearance, and is a defining feature of a well-developed healthy airway epithelium in vivo. Changes in cell number/function, or mucus amount/viscosity are implicated in numerous lung diseases^28,29^, and muco-ciliary clearance often quantified in vitro using fluorescent tracers and/or ciliary beat assays^30,31^. However, it is also possible to observe apical topography and cilia beating *in* vitro, in real-time, using reductionist approaches such as brightfield microscopy and image analysis. For example, patterns may be visualized on the apical surfaces of cultures, which form as a product of mucus secretion, cilia beating and the dimension constraints of the circular culturing well. This can be seen with both epithelial monocultures (Fig. 2A) and NHLF co-cultures (Fig. 2B), where uniform apical secretion patterns were often observed. In contrast, with DHLF co-cultures, the topography of the apical surface secretions appears disordered (Fig. 2C). In order to quantify this visual phenomena, brightfield images were converted, thresholded and analyzed using ImageJ (**Additional file 1**, **Figure S2**), where surface contrast is given as a proxy for apical muco-ciliary uniformity. This is possible due to the contrast in mean grey values between apical secretions and cells, as a percentage of the whole image (Fig. 2D); For epithelial monocultures and NHLF co-cultures, surface contrast is comparable (58.8 ± 2.6% and 60.8 ± 2.3% respectively), whereas DHLF co-cultures have a significantly lower surface contrast (36.6 ± 4.2%). Detailed differences in epithelial cell morphology are visualized in Hematoxylin and eosin (H&E) and immunostained histology sections (Fig. 2E-G) and morphological changes were quantified via image segmentation analysis using ImageJ (**Figure S3**). NHBE monocultures displayed a characteristic pseudostratified columnar epithelial structure and expression of specific epithelial markers including Mucin5B, Mucin 5AC and E-cadherin (Fig. 2E). Interestingly, co-culture with fibroblasts significantly reduced the height of the pseudostratified epithelium, and altered the ratio of Mucin5B/5AC expression, regardless of whether fibroblasts were diseased or healthy (Fig. 2E–K). DHLF co-cultures (Fig. 2H) displayed a significant change in epithelial cell morphology that resembled a squamous phenotype (Fig. 2G), including a reduction in nucleus size, roundness and an increase in nucleus aspect ratio (Fig. 2I–K).

### Characterization of barrier properties and gene expression

**Figure 3:**
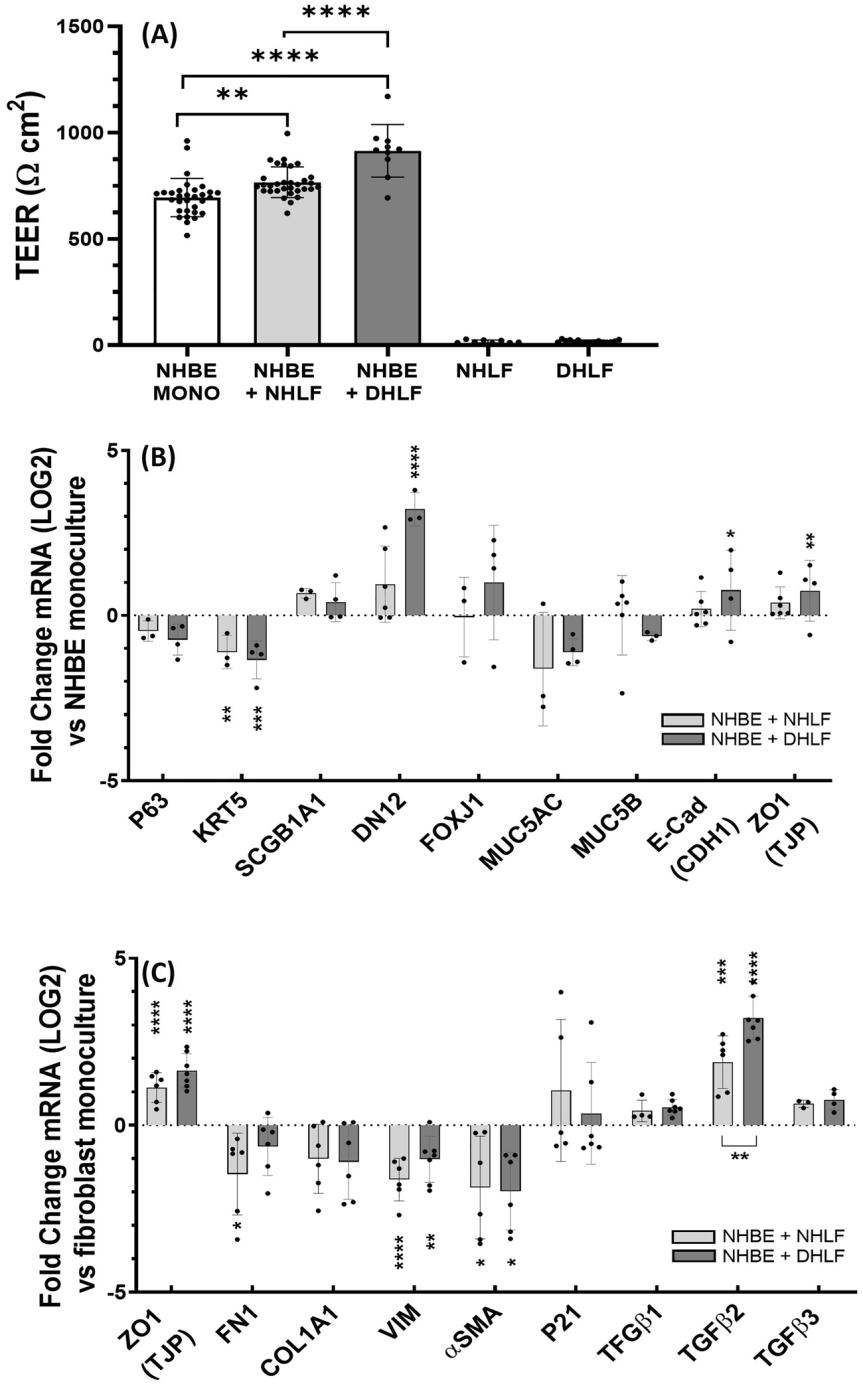
Electrical and mRNA analysis of in vitro cultures. Transepithelial Electrical resistance (TEER) measurements of in vitro cell cultures at day 35 of culture (**A**). Changes in gene expression of co-cultures when normalised and presented as fold log change to epithelial monocultures (**B**) or respective fibroblast monocultures (**C**). Comparisons were performed using a one-way ANOVA with Tukey’s multiple comparisons (****, P < 0.0001; ***, P = 0.0002;**, P = 0.002; *, P = 0.033). n = 2 donors for NHBEs, n = 3 donors for NHLFs and n = 3 donors for DHLFs, N = 4–8 for each condition and presented as the mean ± SD. NHBE = Normal Human Bronchial Epithelial cell; NHLF = Normal Human Lung Fibroblast; DHLF = Diseased Human Lung Fibroblasts.

Transepithelial Electrical Resistance (TEER) is a commonly used method to non-invasively measure in vitro barrier systems. TEER is dependent on the resistance to ion flow through paracellular pathways, including cadherin, adherens and tight-junction proteins, where a higher resistance indicates a stronger barrier^32^. TEER measurements of cultures at day 35 are shown in Fig. 3A; Of note, is that fibroblast monocultures display minimal TEER values, as their physiological role is not of a barrier cell origin^33^. NHBE monocultures display a TEER value of 694.4 ± 90 Ω cm^2^ which is in line with previous reported values of between 600 and 1200 Ω cm^2^ (depending on donor variability)^27,34,35^. However, when grown in co-culture, fibroblasts had a synergistic effect on TEER, reaching significantly higher values than NHBE monoculture in both NHLF (766.9 ± 71.9; **, P = 0.003) and DHLF (914.3 ± 120.4; ****, P =  < 0.001) co-cultures. Interestingly, DHLF co-cultures displayed a small, but significant, increase in TEER value compared to NHLF co-cultures (****, P =  < 0.001). Similarly, mRNA levels of proteins which regulate paracellular transport, namely epithelial Cadherin (CDH1) and the tight junction protein, ZO-1, increase in a culture condition-dependent manner and reach significance in DHLF co-cultures (Fig. 3B; *, P = 0.026; **, P = 0.0061). Ciliated cell mRNA levels (DN12 and FOXJ1) also showed a co-culture dependent increase, reaching significance in DHLF co-cultures, with over a threefold increase compared to NHBE monocultures (****, P =  < 0.001). Mucin mRNA (Fig. 3B) and protein levels (Additional file 1, Figure S4) did not significantly differ between any groups. However, a co-culture dependent reduction in basal cell mRNA levels (P63 and KRT5) were observed, reaching significance in KRT5 expression for both healthy (**, P = 0.0062) and diseased (***, P = 0.009) co-cultures. Next, to assess the effect of epithelial co-culture on fibroblast phenotypes, gene expression is presented as normalized to respective fibroblast monocultures (Fig. 3C). A significant downregulation in mRNA levels of vimentin (****, P =  < 0.001; **, P = 0.0036) and α-smooth muscle actin (α-SMA; *, P = 0.033), were observed in both NHLF and DHLF co-cultures. Additionally, a co-culture dependent increase in mRNA levels of TGFβ-2 (***, P = 0.0003;****, P =  < 0.001) was observed, with an enhanced expression in DHLF vs NHLF co-cultures (**, P = 0.0068).

Overall, the above data demonstrated a significant effect of fibroblast co-culture on epithelial cell phenotypes. This included a reduction in mRNA levels of basal cell markers, epithelium thickness and an increase in barrier integrity (TEER, tight junction and cadherin protein expression). When cultured with healthy fibroblasts, no significant changes in apical topology, cell nucleus morphology or cell density was observed compared to NHBE monoculture. However, when cultured with IPF fibroblasts, highly significant changes were seen in barrier function, cilia mRNA expression and epithelial cell morphology. This included significant alternations in apical topology, epithelium thickness, cell nucleus size/shape and cell density which indicated transition to a squamous phenotype. DHLF co-cultures showed a significant upregulation of TGFβ-2 mRNA levels compared to any other group. Taken together, this data may implicate the role of fibroblast-induced barrier alterations and markers of EMT in the IPF phenotype.

### Platform validation with a proinflammatory stimulus

The transforming growth factor beta (TGFβ) family (isoforms 1–3) are potent regulators of fibrotic processes in mammals and is the main cytokine family upregulated in lung fibrosis and in IPF patient biopsies^24–26^. TGFβ-1 is chosen specifically as a proinflammatory stimulus due to it being the most physiologically abundant and ubiquitously expressed isoform, is readily available and is a well authenticated as a stimulus to induce fibrosis in vitro. However, the effects of TGFβ-1 stimulation, have mostly been characterized in monocultures of fibroblasts or epithelial cells and few studies have looked to evaluate the effect in co-cultures on epithelial barrier properties^7,8,36–39^. Thus, we looked to validate and directly compare, the effect of TGFβ-1 stimulation on co-cultures of healthy epithelial cells grown with either healthy or IPF-derived fibroblasts. Details of primers and probes used for real time q-PCR can be found in Table 1 of the methods section.

**Figure 4:**
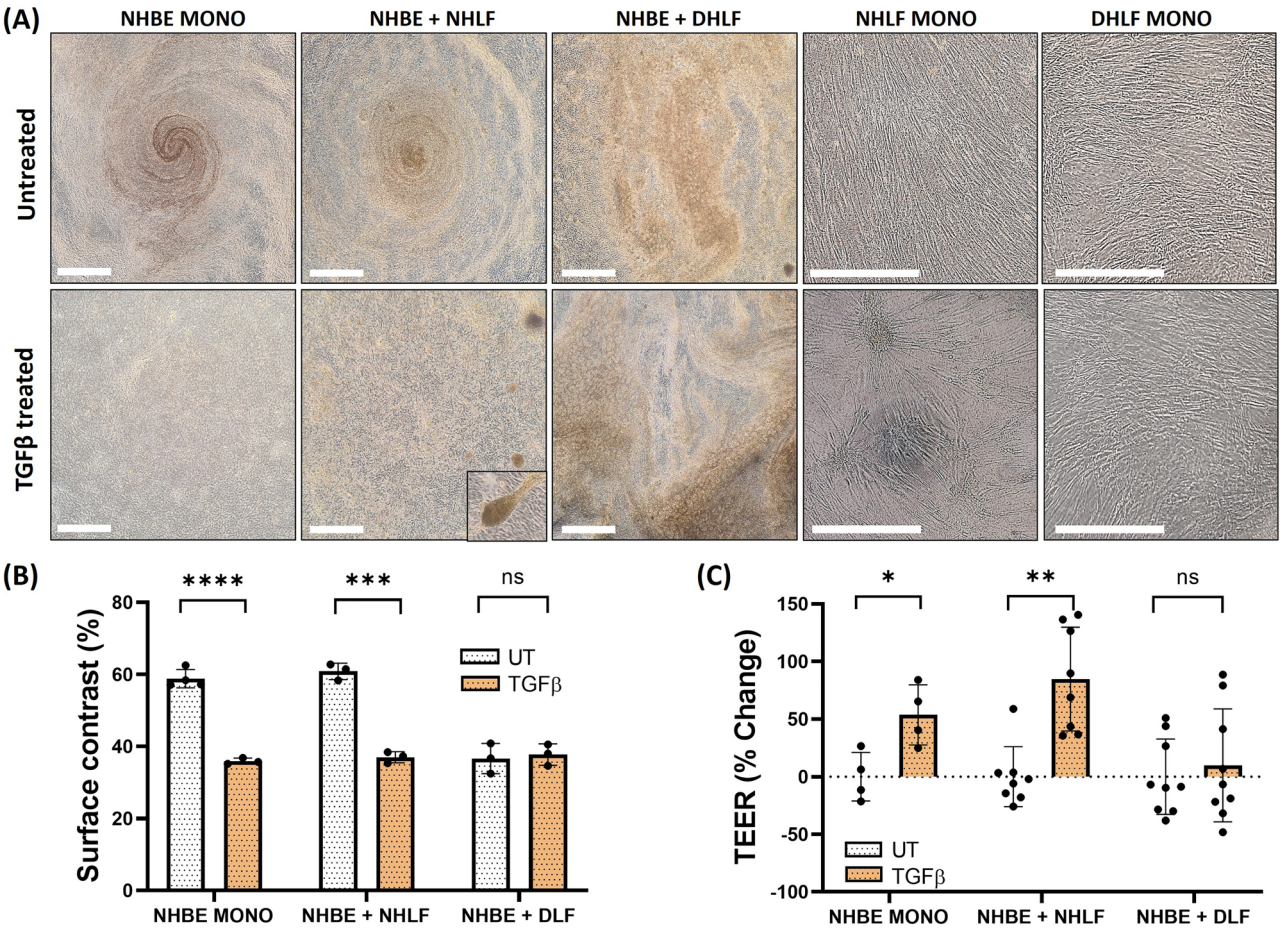
Representative brightfield images of the apical surface of untreated VS TGFβ-1 treated cultures (**A**). Scale bars = 1 mm, inset =  × 3 magnification. Quantification of apical surface contrast (**B**), as a proxy for mucus reflectance, for TGFβ-1 treated cultures. Changes in TEER post-stimulation (**C**) when normalised and presented as percentage change from untreated. Comparisons were performed using multiple T tests with Sidak-Holm multiple comparisons (****, P < 0.0001; ***, P = 0.0002; **, P = 0.002; *, P = 0.033). n = 2 donors for NHBEs, n = 3 donors for NHLFs and n = 3 donors for DHLFs, N = 4–8 for each condition and presented as the mean ± SD. NHBE = Normal Human Bronchial Epithelial cell; NHLF = Normal Human Lung Fibroblast; DHLF = Diseased Human Lung Fibroblasts ; UT = untreated.

On day 35, co-cultures were treated basolaterally with activated human TGFβ-1 (1 ng/ml) for one week. The concentration of TGFβ-1 was determined based on previous literature, where doses of between 1–10 ng/ml have been authenticated and extensively used to induce EMT in cell monocultures and organoids^7,8,36–40^. In NHBE monocultures and NHLF co-cultures, treatment significantly disrupted apical muco-ciliary architecture, whereas DHLF co-cultures appeared unaffected (Fig. 4A). Additionally, treatment induced the formation of fibrotic-like foci in NHLF but not DHLF cultures (Fig. 4A). As aforementioned, brightfield images of the apical surface of cultures can be analyzed, contrasted and quantified using ImageJ to give surface contrast values as a proxy for apical muco-ciliary uniformity (Fig. 4B). A significant reduction is observed for NHBE monocultures (****, P < 0.001) and NHLF co-cultures (***,P = 0.002) but not DHLF co-cultures (P = 0.73). When quantifying TGFβ-1-induced changes in epithelial barrier resistance, data are presented as normalized to pre-treatment values. This is due to the donor-to-donor and well-to-well variability, so often seen in cell culture. Thus, to remove this variability, wells are matched (pre-to-post treatment) and the overall percentage change compared. TGFβ-1 treatment resulted in a significantly increased TEER for NHBE monocultures (53.6 ± 26.1%, *, P = 0.037) and NHLF co-cultures (84.7 ± 45.0%, **, P = 0.001) but not DHLF (9.88 ± 49.0%, P = 0.62) co-cultures (Fig. 4C).

**Figure 5:**
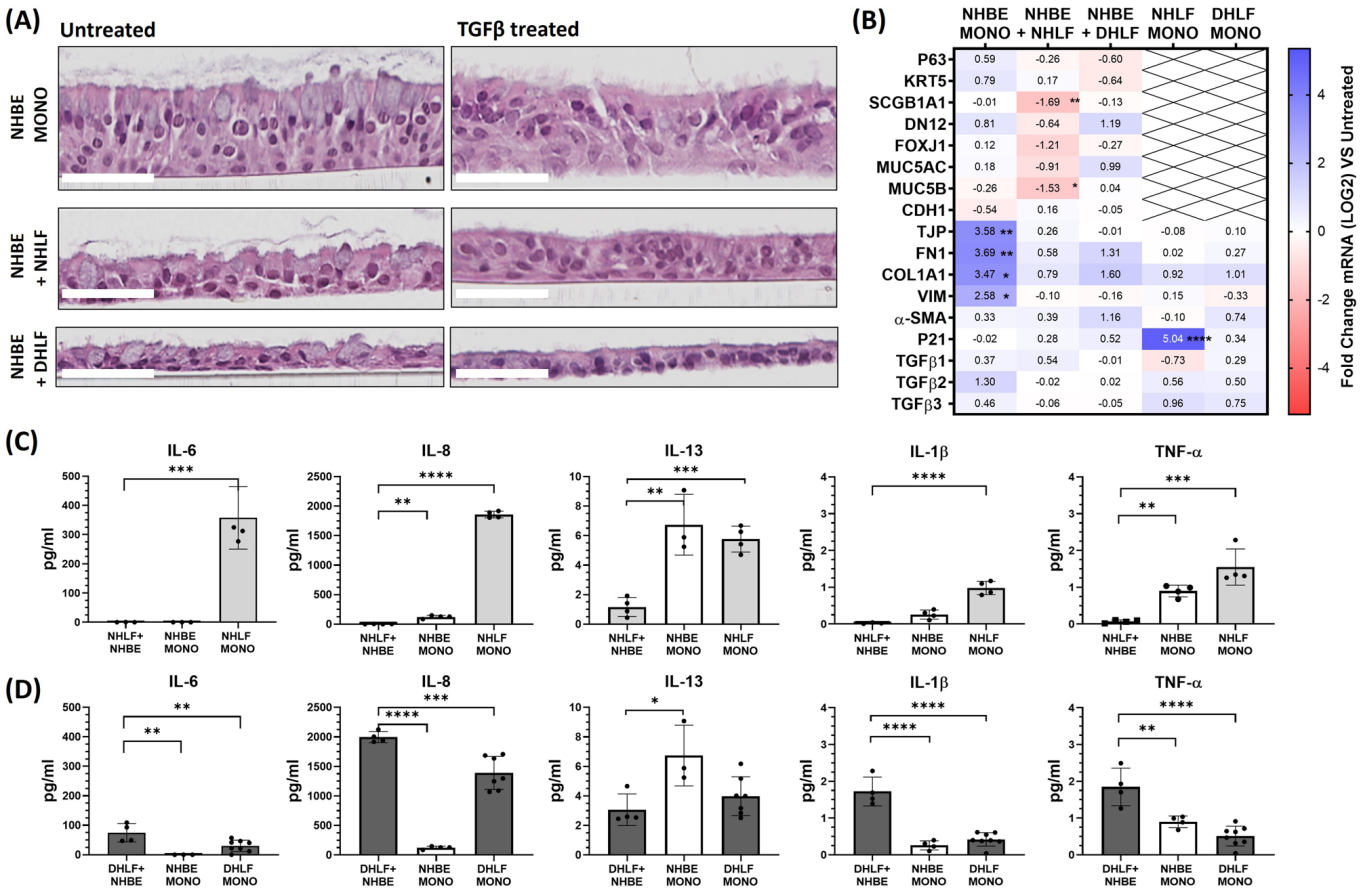
Representative H&E stained histology slices of untreated and TGFβ-1 treated cultures (**A**). Scale bars = 50 µm. Change in gene expression of TGFβ-1 treated cultures (**B**). Data are normalised and presented as fold log change to respective untreated groups, comparisons performed using multiple T tests with Sidak-Holm multiple comparisons. Change in cytokine release of TGFβ-1 treated cultures for healthy (**C**) and diseased (**D**) cultures. Data are presented in pg/ml with comparisons made using a one-way ANOVA with Tukey’s multiple comparisons. (****, P < 0.0001; *** P, = 0.0002;**, P = 0.002; *, P = 0.033). n = 2 donors for NHBEs, n = 3 donors for NHLFs and n = 3 donors for DHLFs, N = 4–8 for each condition and presented as the mean ± SD. NHBE = Normal Human Bronchial Epithelial cell; NHLF = Normal Human Lung Fibroblast; DHLF = Diseased Human Lung Fibroblasts ; UT = untreated.

In terms of cell morphology, H&E stained histology sections offer further insights (Fig. 5A**).** NHBE monocultures show the greatest TGFβ-1-induced alterations with significant reductions in epithelium height, cell polarity, nucleus size/roundness and changes in muco-ciliary phenotype (e.g. height and thickness) compared to NHLF co-cultures (Fig. 5A). DHLF co-cultures, on the other hand, did not display any significant alterations in cell morphology, height or muco-ciliary architecture when treated with TGFβ-1. Levels of mRNA markers implicated in Epithelial-to-Mesenchymal-Transition (EMT), were significantly upregulated in TGFβ-1-treated NHBE monocultures (Fig. 5B); ECM-associated mRNA levels of fibronectin (FN1) and Collagen 1 (COL1) were upregulated over threefold (**, p = 0.010; *, P = 0.033) and Vimentin (VIM) over twofold (*, P = 0.033). In contrast, mRNA levels of EMT markers in NHLF co-cultures did not change significantly. NHLF co-cultures, however, showed a down regulation of club cell (SCGB1A1; *, P = 0.0018) and goblet cell (MUC5B;*, P = 0.0050) mRNA levels. Similarly to the visual phenotypes observed, no significant changes in gene expression were apparent for TGFβ-1 treated DHLF monocultures or co-cultures. In fibroblast monocultures, NHLFs showed treatment-induced upregulation of senescence markers (****, P =  < 0.001).

Although gene expression analysis can give important insights into the phenotypic changes observed, it is important to show how these results influence protein synthesis. To this end, TGFβ-1-induced pro-inflammatory cytokine release (IL-6, IL-8, IL-13, IL1-β and TNF-α) was quantified by sampling basal supernatants. A detailed list of analytes and typical detection limits can be found in Table 2 of the methods section. Compared to untreated controls, TGFβ-1 stimulation significantly enhanced cytokine release across all groups (Additional file 1, Figure S5) with the exception of NHLF co-cultures which showed a significantly large reduction in all pro-inflammatory cytokines. The protective effect of NHLF co-cultures against TGFβ-1-induced inflammation is highlighted when compared to NHBE and NHLF monocultures (Fig. 5C). NHLF co-cultures showed a significant reduction in IL-6 (***, P = 0.005), IL-8 (****, P =  < 0.0001), IL-13 (***, P = 0.007), IL-1β (****, P =  < 0.0001) and TNF-α (***, P = 0.0001) when compared to NHLF monoculture and a significant reduction in IL-8 (**, P = 0.0019), IL-13 (**, P = 0.0014) and TNF-α (**, P = 0.0019) when compared to NHBE monoculture (Fig. 5C). In contrast, when grown with diseased fibroblasts, an agonistic effect on TGFβ-1-induced inflammation was observed in co-cultures. This is highlighted when compared to NHBE and DHLF monocultures (Fig. 5D); DHLF co-cultures showed a significant increase in IL-6 (***, P = 0.0098), IL-8 (***, P = 0.0009), IL-1β (****, P =  < 0.0001) and TNF-α (***, P =  < 0.0001) when compared to DHLF monoculture and a significant increase in IL-6 (**, P = 0.0012), IL-8 (****, P =  < 0.0001), IL-1β (****, P =  < 0.0001) and TNF-α (**, P = 0.0022) when compared to NHBE monoculture. IL-13 was the only exception, in which NHBE monocultures showed the largest amount (6.74 pg/ml) released when compared to any other culture group (**, P = 0.0014 vs NHLF, Fig. 5C**;** *, P = 0.0112 vs DHLF, Fig. 5D). This is in line with previous reports implicating the specific role of epithelial IL-13 in enhancing inflammation, remodeling and EMT in response to TGFβ-1^41,42^.

## Discussion

With respect to clinical relevance and translatability, the in vitro model used here mimics the in vivo conditions of the airway epithelial-stromal layer more accurately than routine monocultures of fibroblasts or bronchial epithelial cells. Previously, the effect of direct epithelial-fibroblastic co-culture on the structure, function and barrier integrity of bronchial epithelial cell in the context of IPF models, has been poorly described. Conventionally co-cultures grown fibroblasts indirectly on culture plastic, and their temporary effects on epithelial cells inserted above on hanging inserts are measured. However, our model addresses a significant gap in the in vitro literature, where the effects of direct contact/cross-talk may be investigated. Although the use of IPF-derived epithelial cells would better reflect the diseased environment, and would provide further insights into IPF dysregulated cross-talk, they are extremely difficult to access. Thus, this model provides an alternative method to generate IPF-related epithelial phenotype with mixed donors and is an is an important first step in generating a more biomimetic model.

The model was successively used to study interactions between bronchial epithelial cells from healthy donors and fibroblasts from either healthy or IPF donors. Contrary to what was expected, the effect of co-culture decreased epithelial thickness and stratification, irrespective of whether fibroblasts were from healthy of IPF donors. Additionally, immunostaining revealed that the ratio of MUC5AC to MUC5B expression was altered in co-cultures, skewed in the direction of MUC5B. A reduction in epithelial height, thickness and stratification is a phenotype often observed in lung diseases such as COPD and cigarette-smoke epithelial injury^17,22,23^. Furthermore, overexpression and genetic polymorphisms of MUC5B are known risk factors associated with IPF^49,50^. In DHLF co-cultures, a reduction in epithelial height was the greatest and EMT-related morphological changes (nucleus elongation and size reduction) reached significance compared to any other group. This phenotype is a likely consequence of DHLF-induced EMT, as reported previously^30,31^. For the phenotype observed with NHLF co-cultures, a possible explanation is that fibroblasts induce the de-differentiation of epithelial tissue and that NHLF co-cultures actually represent an intermediate EMT phenotype. Another possibility may lie within how cells are routinely cultured on cell culture plastic, prior to seeding. It has been shown previously, for example, that culturing fibroblast on hard surfaces^45^ or at a high densities^6^ induce phenotypic transitions towards FMT and IPF-related cell populations. Thus, whether healthy fibroblasts retain their true in vivo phenotype when cultured over time, remains a controversial topic. To minimize this effect however, all cells were seeded onto collagen coated membrane at no higher than passage one and epithelial cells were differentiated at ALI for 28 days prior to seeding fibroblasts. Additionally, three healthy and three IPF donors were used which increases our level of confidence that any differences observed are due to differences in disease state, rather than simply donor to donor variability. Techniques such as single cell RNA sequencing should be utilized in future studies to elucidate the causal mechanisms behind this co-culture dependent alteration in epithelial phenotype.

Another characteristic feature of other lung diseases, such as asthma^20,21^ and COPD^22,23^, is epithelial barrier dysfunction. However, the pathogenic implications of barrier (dys)function in the progression of IPF is ill defined. TEER is a commonly used method to determine the quality of differentiated epithelial airway development and barrier integrity^32^. Contrary to what one might expect, given the lower stratification of epithelial layers, our study demonstrated that fibroblast co-culture had a synergistic effect on TEER, reaching the highest values when cultured with DHLFs. As TEER is dependent on the resistance to ion flow through paracellular pathways, the co-culture dependent increase in TEER may be influenced by the expression of proteins that regulate paracellular barrier permeability. Supportive of this hypothesis, it the increase in mRNA levels of the tight junction proteins CDH1 and ZO-1 with co-culture. Previously, reported data also demonstrate an increased ZO-1 expression in *ex-vivo* IPF bronchiolar biopsies^51^ and in vitro bronchial co-culture models^37,52,53^. Additionally, a decrease in epithelial height/stratification corresponds to an increase in epithelial tissue density, when co-cultured with fibroblasts. Thus, an increase in tissue density may correlate to the increase in resistance (TEER), a relationship previously described^54^.

Functional muco-ciliary clearance mechanisms are another defining feature of a well-developed healthy airway epithelium with changes in cell number/function, or mucus amount/viscosity implicated in numerous lung diseases^28,29^. Qualitatively, the synergistic functions of mucus secretion, cilia beats and the circular dimensions of the culture surface gave rise to apical surface patterns which could be observed clearly using brightfield microscopy. Epithelial cell surface topography was uniform and consistent between NHBE monocultures and NHLF cocultures. However, when grown with DHLF the apical topography was disrupted and heterogenous. Using image processing, this visual phenomena was quantified using surface contrast and given as a proxy for muco-ciliary uniformity. Together with H&E stains, taken of the same samples, a significant reduction in epithelial muco-ciliary uniformity and cilia projections were observed in the DHLF co-culture condition. Additionally, over a threefold increase in ciliated cell mRNA markers were apparent, while mucin mRNA and protein levels did not differ between any groups. Changes in cilia, rather than mucin function, therefore, may contribute to the aberrant surface topology observed. It is worth noting however that cilia function is determined here through imaging processing of fixed samples, brightfield captures and mRNA analysis. Complementary functional assays may be used in future studies to assess if changes in gene/protein expression correlate to a cell functionality e.g. cilia beat/motility assays or mucin viscosity. Interestingly, basal cell markers were downregulated in co-cultures, irrespective of fibroblast origin. As stem cells of the lung epithelium, basal cells may be associated with normal wound healing processes, proliferation, injury or EMT^28^. Future studies could look to explore specific downstream pathways and other basal cell markers e.g. KRT14, SOX2, TP63 to shed light on any pathogenic implications. In terms of fibroblast specific markers, vimentin and α-SMA mRNA levels were downregulated. Additionally, mRNA levels of TGFβ-2, were significantly upregulated. Although vimentin and α-SMA are markers of FMT^52^, and TGFβ-2 plays a pathological role in lung fibrosis^33,55,56^, analysis at the protein and functional level should be conducted in future studies, to define the implications of these changes. Nonetheless, the significant quantitative and qualitive differences observed between each co-culture condition, with respect to epithelial and fibroblast monocultures, highlights the utility of this platform to be used for interrogating the effects of direct epithelial-fibroblastic crosstalk in the development of IPF phenotypes. We hope this platform can therefore be used for identifying and validating novel targets, that would otherwise be missed in routine monoculture or indirect co-culture studies.

Next, we looked to validate and directly compare, the effect of TGFβ-1 stimulation on co-cultures. TGFβ-1 is the most widely authenticated stimulus used to induce fibrosis in in vitro fibroblast and epithelial monocultures, however, few studies have looked to evaluate if direct cross-talk between the cell populations influence the TGFβ-1 response. Additionally, the majority of studies assess the effect of TGFβ-1 by means of cytokine, protein and gene expression but the effect on epithelial barrier properties in co-cultures is poorly described^7,8,33,37–39^. In this study, imaging techniques revealed a significant disruption of epithelial muco-ciliary uniformity in TGFβ-1 treated NHBE monocultures and NHLF co-cultures, that was not apparent in DHLF co-cultures. Similarly, TGFβ-1 induced the formation of fibrotic-like foci in NHLF co-cultures and monocultures that was not observed in DHLF co-cultures. This suggests that exogenous TGFβ-1 may be producing less of a stimulatory effect on observational phenotypes in IPF fibroblasts, potentially due to the enhanced pro-inflammatory environment intrinsically produced by diseased fibroblasts. This is supported by the significantly higher levels of TGFβ-1 and -2 mRNA in untreated DHLF compared to NHLF monocultures (Additional file 1, Figure S6) and, when stimulated, release of all cytokines tested are significantly lower in DHLF (Additional file 1, Figure S6)**.** Additionally, as shown in Fig. 3C, levels of TGFβ isoforms are higher in DHLF than NHLF co-cultures prior to stimulation.

TGFβ-1 significantly increased TEER values in NHBE monocultures and NHLF co-cultures but not DHLF co-cultures. Although in vitro models of other lung disease, such as asthma^20,21^ and COPD^22,23^, report reductions in epithelial integrity compared to healthy controls, actual values of TEER are wildly variable. In addition, the majority of studies fail to match pre-post treatment TEER values, meaning that baseline well-to-well and donor-to-donor variability is not accounted for. In a primary in vitro model of asthma, which directly compared the effect of TGFβ-1 stimulation on co-cultures of bronchial epithelial and fibroblast cells, a protective effect of fibroblasts against TGFβ-1-induced EMT and barrier integrity was observed^52^. Additionally, healthy and DHLF co-cultures gave differential pro-fibrotic responses in response to TGFβ-1^52^. However, few studies have looked to characterize the pathogenic implications of epithelial barrier (dys)function specifically in the progression of IPF. This is partly due to the traditional use of non-barrier forming cells such as fibroblast or alveoli (type 2) cells and/or the inability to source IPF derived epithelial cells. In the context of this study, which specifically looks at the effect of IPF fibroblasts on NHBEs phenotypes, TEER values not defined in literature. Future work, beyond the scope of this study, should look to source IPF derived epithelial cells (if possible). In this way, the direct co-culture of IPF epithelial and fibroblast cells can then be compared to the in vitro IPF epithelial monocultures^9,45,57^ to fully elucidate the cell- and disease-specific contribution to epithelial barrier resistance.

In terms of epithelial thickness, stratification, apical topology and nucleus shape, H&E images revealed substantial disruptions in TGFβ-1 treated NHBE monocultures. Additionally, mRNA markers of EMT were highly and significantly upregulated, which is in line with previous reports of TGFβ-1 treated bronchial epithelial cell lines and primary cells^7,8,36^. In contrast, epithelial cell morphology and mRNA levels of EMT markers, in NHLF co-cultures, remained analogous to pretreatment conditions. This may suggest a protective role of healthy fibroblast against TGFβ-1-induced EMT, which is a finding previously reported in a primary co-culture model of the epithelial-mesenchymal trophic unit^52^. Furthermore, quantification of TGFβ-1-induced pro-inflammatory cytokine release conferred a protective effect of NHLF co-cultures against TGFβ-1-induced inflammation; When stimulated and compared to either monoculture alone, NHLF cocultures released significantly less IL-6, IL-8, IL-13, IL1-β and TNF-α. Additionally, the significantly enhanced TGFβ-1 induced inflammatory response of healthy vs diseased fibroblasts monocultures (Additional file 1, Figure S7) was nullified when co-cultured with NHBEs. However it is also worth considering the length and administration of treatment. For example, since TGFβ-1 is given basally, stimulation is limited by diffusional distance and thickness of cell layers. In the case of co-cultures, where there is an additional layer of cells, extra time and/or a higher concentration of treatment may be needed in order to exert the same effect as in monocultures. It may be that here, NHLF co-cultures present an intermediate phenotype, prior the EMT. Indeed, there were some significant alterations in mRNA expression of club cell and goblet cell markers which may indicate the initiation of a fibrotic phenotype^58^. Future studies should investigate longer treatment times and any time-dependent changes in transitional markers. There was no significant effect of TGFβ-1 stimulation on phenotypic transitions in DHLF monocultures or co-cultures, compared to their respective untreated condition. However, DHLF co-cultures displayed an agonistic effect on TGFβ-1-induced inflammation, with increases in IL-6, IL-8 , IL-1β. IL-1β TNF-α compared to DHLF or NHBE monoculture. Interestingly, NHLF monocultures showed a treatment induced upregulation of senescent mRNA markers. Although cellular senescent pathways have been implicated in IPF pathology and in fibroblasts isolated from IPF patients^59,60^, other studies report TGFβ-induced FMT alterations in fibroblast monocultures^7–9^. One explanation for the lack of reproducibility between experimental studies is changes to environmental conditions or configurations. As aforementioned, whether healthy fibroblasts retain their true in vivo phenotype when cultured over time, also remains a controversial topic. This highlights the need for models which better reflect the native tissue ECM environment, which we hope this study will provide a starting point for. Nonetheless, TGFβ-1 stimulation gave rise to differential responses between each culture condition and holds implications for any translation conclusions drawn from studies were cells are grown in monoculture or in indirect contact.

In summary, analysis at the gene and secreted protein level may indicate a protective effect of healthy fibroblasts and NHBEs when they were co-cultured. TGFβ-induced EMT, FMT and inflammatory responses were significantly blunted compared to either cell type grown as a monoculture. In contrast, DHLF co-cultures demonstrated an enhanced inflammatory response without any significant phenotypic transitions. To elucidate casual mechanisms of co-culture however, future studies may wish to evaluate protein levels and/or single cell RNA sequencing. The differential responses between culture groups highlight the importance of epithelial-fibroblastic cross-talk in the initiation of inflammatory pathways and should be taken into consideration when developing in vitro models of IPF, especially for therapeutic and drug discovery applications. Based on the overall results reported, co-culture with either NHLFs or DHLFs may both be useful models in generating diseased epithelial phenotypes while overcoming the scarcity in obtaining primary IPF epithelial cells.

Next steps, beyond the scope of this initial study, should look to build upon the complexity of the model by including additional cell types implicated in IPF pathogenesis. For example, it is known that both innate and regulatory immune cells play an important role in IPF progression^46–48^. Thus, future studies could look to improve the clinical relevance of the model by including direct and/or indirect immune cells cross-talk. Additionally, the incorporation of small airway or alveoli cells e.g. Human Type II Alveolar Epithelial Cells (PAEpiC2) could be compared and contrasted to the bronchial epithelial model generated here. Indeed, Type II Alveolar Epithelial Cell injury and bronchiolization of the alveoli epithelium are known to occur in the initial stages of IPF development^43–45^. Thus, comparisons of co-cultures generated with anatomically distinct epithelial cell populations (bronchial or type II alveolar epithelial cells) would provide a more comprehensive insight into the involvement of fibroblasts in the development of IPF. Finally, since the pathogenesis of IPF is complex and mechanisms extend beyond EMT/inflammation^1,10,47,61^, future studies may wish to stimulate cultures with a range of compounds e.g. viruses, particles or chemicals to comprehensively evaluate the model for their desired research question.

## Conclusions

The majority of previous in vitro IPF studies have looked to characterize cellular changes involved in disease progression using reductionist models such as monocultures of fibroblasts or epithelial cells. Although this step is essential in defining the unique role each cell plays, the effect of the physiological (in vivo) environment e.g. multiple cell types, 3D structure is nullified. We have shown that the effect of co-culturing epithelial cells in a quasi-3D model, with healthy or diseased fibroblasts, had a significant effect on barrier properties and gene expression of markers associated with a healthy epithelial phenotype. Additionally, both co-culture and monoculture groups gave differential responses to TGFβ-1, a well authenticated pro-inflammatory stimulus used model IPF in vitro. This finding is important to consider when interpreting monoculture studies for therapeutic discovery and is an important step in development of more biomimetic models of IPF. Future studies could look to improve the biomimicry of the model further with the use of additional cell types e.g. immune cells, alveolar type II cells, IPF-derived HBEs in response to inflammatory stimuli. Culturing cells in a more physiological 3D and biomechanical environment, such as hydrogels, scaffolds or microfluidics will also facilitate developmental processes and response to drug challenge^11–14^. Additionally, the development of automated in-line sensors to monitor the development and progression of disease phenotypes will greatly improve our understanding of, and ability to interrogate, these 3D systems. Indeed, future work aims to adapt our previously published electronic transmembrane device^62^ for lung-on-chip applications.

### Supplementary Information

Supplementary Information.

## Data Availability

The datasets used and/or analyzed during the current study are available from the corresponding author on reasonable request.

## Abbreviations

2D: Two dimensional
3D: Three dimensional
ALI: Air liquid interface
COPD: Chronic obstructive pulmonary disorder
DHLF: Diseased human lung fibroblasts
EMT: Epithelial-to-mesenchymal transition
FITC: Fluorescein isothiocyanate
FMT: Fibroblast-to-myofibroblast transition
IL: Interleukin
IPF: Idiopathic pulmonary fibrosis
NHBE: Normal human bronchial epithelial cell
NHLF: Normal human lung fibroblast
TEER: Transepithelial electrical resistance
TGF: Transforming growth factor
TNF: Tumor necrosis factor

## References

[1] Martinez FJ, Collard HR, Pardo A, Raghu G, Richeldi L, Selman M (2017). Idiopathic pulmonary fibrosis. Nature Reviews Disease Primers.

[2] Mei Q, Liu Z, Zuo H, Yang Z (2022). Idiopathic pulmonary fibrosis: An update on pathogenesis. Front. Pharmacol..

[3] Marijic P, Schwarzkopf L, Schwettmann L, Ruhnke T, Trudzinski F, Kreuter M (2021). Pirfenidone vs. nintedanib in patients with idiopathic pulmonary fibrosis: A retrospective cohort study. Respir. Res..

[4] Wollin L, Maillet I, Quesniaux V, Holweg A, Ryffel B (2014). Antifibrotic and anti-inflammatory activity of the tyrosine kinase inhibitor nintedanib in experimental models of lung fibrosis. J. Pharmacol. Exp. Ther..

[5] Schruf E, Schroeder V, Kuttruff CA, Weigle S, Krell M, Benz M (2019). Human lung fibroblast-to-myofibroblast transformation is not driven by an LDH5-dependent metabolic shift towards aerobic glycolysis. Respir. Res..

[6] Doolin MT, Smith IM, Stroka KM. Fibroblast to myofibroblast transition is enhanced by increased celldensity. Mol. Biol. Cell. 2021;32(22). 10.1091/mbc.E20-08-0536 PMC8694087 34731044

[7] Doerner AM, Zuraw BL (2009). TGF-β1induced epithelial to mesenchymal transition (EMT) in human bronchial epithelial cells is enhanced by IL-1β but not abrogated by corticosteroids. Respir. Res..

[8] Kamitani S, Yamauchi Y, Kawasaki S, Takami K, Takizawa H, Nagase T (2011). Simultaneous stimulation with TGF-β1 and TNF-α induces epithelial mesenchymal transition in bronchial epithelial cells. Int. Arch. Allergy Immunol..

[9] Stancil IT, Michalski JE, Davis-Hall D, Chu HW, Park J-A, Magin CM, et al. Pulmonary fibrosis distal airway epithelia are dynamically and structurally dysfunctional. Nat. Commun. 2021;12(4566). 10.1038/s41467-021-24853-8 PMC8316442 34315881

[10] Selman M, Pardo A. Idiopathic pulmonary fibrosis: An epithelial/fibroblastic cross-talk disorder. Respir Res. 2002;3(1). 10.1186/rr175 PMC64814 11806838

[11] O’leary C, Cavanagh B, Unger RE, Kirkpatrick J, O’dea S, O’brien FJ, et al. The development of a tissue-engineered tracheobronchial epithelial model using a bilayered collagen-hyaluronate scaffold. 2016; 10.1016/j.biomaterials.2016.01.065 26871888

[12] Albers S, Thiebes AL, Gessenich KL, Jockenhoevel S, Cornelissen CG (2016). Differentiation of respiratory epithelium in a 3-dimensional co-culture with fibroblasts embedded in fibrin gel. Multidiscip. Respir. Med..

[13] Burkhanova U, Harris A, Leir SH (2022). Enhancement of airway epithelial cell differentiation by pulmonary endothelial cell co-culture. Stem Cell Res..

[14] Barron SL, Saez J, Owens RM (2021). In vitro models for studying respiratory host-pathogen interactions. Adv Biol..

[15] Pain M, Bermudez O, Lacoste P, Royer PJ, Botturi K, Tissot A (2014). Tissue remodelling in chronic bronchial diseases: From the epithelial to mesenchymal phenotype. Eur. Respir. Rev..

[16] Carlier FM, de Fays C, Pilette C (2021). Epithelial barrier dysfunction in chronic respiratory diseases. Front. Physiol..

[17] Ghosh B, Nishida K, Chandrala L, Mahmud S, Thapa S, Swaby C, et al. Epithelial plasticity in COPD results in cellular unjamming due to an increase in polymerized actin. J Cell Sci. 2022;135(4). 10.1242/jcs.258513 PMC8919336 35118497

[18] Ghosh B, Reyes-Caballero H, Akgün-Ölmez SG, Nishida K, Chandrala L, Smirnova L, et al. Effect of sub-chronic exposure to cigarette smoke, electronic cigarette and waterpipe on human lung epithelial barrier function. BMC Pulm Med. 2020;20(1). 10.1186/s12890-020-01255-y PMC7425557 32787821

[19] Ghosh B, Loube J, Thapa S, Ryan H, Capodanno E, Chen D, et al. Loss of E-cadherin is causal to pathologic changes in chronic lung disease. Commun Biol. 2022;5(1). 10.1038/s42003-022-04150-w PMC9617938 36309587

[20] Wawrzyniak P, Wawrzyniak M, Wanke K, Sokolowska M, Bendelja K, Rückert B (2017). Regulation of bronchial epithelial barrier integrity by type 2 cytokines and histone deacetylases in asthmatic patients. J. Allergy Clin. Immunol..

[21] Xiao C, Puddicombe SM, Field S, Haywood J, Broughton-Head V, Puxeddu I (2011). Defective epithelial barrier function in asthma. J. Allergy Clin. Immunol..

[22] Milara J, Peiró T, Serrano A, Cortijo J (2013). Epithelial to mesenchymal transition is increased in patients with COPD and induced by cigarette smoke. Thorax..

[23] Roscioli E, Hamon R, Lester SE, Jersmann HPA, Reynolds PN, Hodge S (2018). Airway epithelial cells exposed to wildfire smoke extract exhibit dysregulated autophagy and barrier dysfunction consistent with COPD. Respir. Res..

[24] Bergeron A, Soler P, Kambouchner M, Loiseau P, Milleron B, Valeyre D (2003). Cytokine profiles in idiopathic pulmonary fibrosis suggest an important role for TGF-β and IL-10. Eur. Respir. J..

[25] Prasse A, Carleo A, Jaeger B, Schupp J, Rottoli P, Wuyts W (2018). BAL cell transcriptome predicts survival in IPF and can be used to gauge and model treatment effects interfering with the TGF-beta pathway. Eur. Respir. J..

[26] Khalil N, Parekh TV, O’Connor R, Antman N, Kepron W, Yehaulaeshet T (2001). Regulation of the effects of TGF-β1 by activation of latent TGF-β1 and differential expression of TGF-β receptors (TβR-I and TβR-II) in idiopathic pulmonary fibrosis. Thorax..

[27] Pezzulo AA, Starner TD, Scheetz TE, Traver GL, Tilley AE, Harvey BG, et al. The air-liquid interface and use of primary cell cultures are important to recapitulate the transcriptional profile of in vivo airway epithelia. Am J Physiol Lung Cell Mol Physiol. 2011;300(1). 10.1152/ajplung.00256.2010 PMC3023285 20971803

[28] Ridley C, Thornton DJ (2018). Mucins: The frontline defence of the lung. Biochemical Society Transactions.

[29] Tilley AE, Walters MS, Shaykhiev R, Crystal RG (2015). Cilia dysfunction in lung disease. Annu. Rev. Physiol..

[30] Jing JC, Chen JJ, Chou L, Wong BJF, Chen Z (2017). Visualization and detection of ciliary beating pattern and frequency in the upper airway using phase resolved doppler optical coherence tomography. Sci. Rep..

[31] Katoh TA, Ikegami K, Uchida N, Iwase T, Nakane D, Masaike T (2018). Three-dimensional tracking of microbeads attached to the tip of single isolated tracheal cilia beating under external load. Sci. Rep..

[32] Srinivasan B, Kolli AR, Esch MB, Abaci HE, Shuler ML, Hickman JJ (2015). TEER measurement techniques for in vitro barrier model systems. J. Lab. Autom..

[33] Plikus MV, Wang X, Sinha S, Forte E, Thompson SM, Herzog EL (2021). Fibroblasts: Origins, definitions, and functions in health and disease. Cell..

[34] Lin H, Li H, Cho HJ, Bian S, Roh HJ, Lee MK (2007). Air-liquid interface (ALI) culture of human bronchial epithelial cell monolayers as an in vitro model for airway drug transport studies. J. Pharm. Sci..

[35] Rayner RE, Makena P, Prasad GL, Cormet-Boyaka E (2019). Optimization of normal human bronchial epithelial (NHBE) cell 3D cultures for in vitro lung model studies. Sci. Rep..

[36] Kim BN, Ahn DH, Kang N, Yeo CD, Kim YK, Lee KY (2020). TGF-β induced EMT and stemness characteristics are associated with epigenetic regulation in lung cancer. Sci. Rep..

[37] Epa AP, Thatcher TH, Pollock SJ, Wahl LA, Lyda E, Kottmann RM (2015). Normal human lung epithelial cells inhibit transforming growth factor-β induced myofibroblast differentiation via prostaglandin E2. PLoS One..

[38] Rangarajan S, Kurundkar A, Kurundkar D, Bernard K, Sanders YY, Ding Q (2016). Novel mechanisms for the antifibrotic action of nintedanib. Am. J. Respir. Cell. Mol. Biol..

[39] Stratmann AT, Fecher D, Wangorsch G, Göttlich C, Walles T, Walles H (2014). Establishment of a human 3D lung cancer model based on a biological tissue matrix combined with a Boolean in silico model. Mol. Oncol..

[40] Ptasinski V, Monkley SJ, Öst K, Tammia M, Alsafadi HN, Overed-Sayer C, et al. Modeling fibrotic alveolar transitional cells with pluripotent stem cell-derived alveolar organoids. Life Sci. Alliance. 2023;6(8). 10.26508/lsa.202201853 PMC10213712 37230801

[41] Malavia NK, Mih JD, Raub CB, Dinh BT, George SC (2008). IL-13 induces a bronchial epithelial phenotype that is profibrotic. Respir. Res..

[42] Seibold MA (2018). Interleukin-13 stimulation reveals the cellular and functional plasticity of the airway epithelium. Ann. Am. Thorac. Soc..

[43] Parimon T, Yao C, Stripp BR, Noble PW, Chen P (2020). Alveolar epithelial type ii cells as drivers of lung fibrosis in idiopathic pulmonary fibrosis. Int. J. Mol. Sci..

[44] Stancil IT, Michalski JE, Davis-Hall D, Chu HW, Park JA, Magin CM (2021). Pulmonary fibrosis distal airway epithelia are dynamically and structurally dysfunctional. Nat. Commun..

[45] Schruf E, Schroeder V, Le HQ, Schönberger T, Raedel D, Stewart EL (2020). Recapitulating idiopathic pulmonary fibrosis related alveolar epithelial dysfunction in a human iPSC-derived air-liquid interface model. FASEB J..

[46] van Geffen C, Deißler A, Quante M, Renz H, Hartl D, Kolahian S (2021). Regulatory immune cells in idiopathic pulmonary fibrosis: Friends or foes?. Front. Immunol..

[47] Desai O, Winkler J, Minasyan M, Herzog EL. The role of immune and inflammatory cells in idiopathic pulmonary fibrosis. Front. Med. 2018;5(MAR). 10.3389/fmed.2018.00043 PMC5869935 29616220

[48] Kolahian S, Fernandez IE, Eickelberg O, Hartl D (2016). Immune mechanisms in pulmonary fibrosis. Am. J. Respir. Cell Mol. Biol..

[49] Hancock LA, Hennessy CE, Solomon GM, Dobrinskikh E, Estrella A, Hara N (2018). Muc5b overexpression causes mucociliary dysfunction and enhances lung fibrosis in mice. Nat. Commun..

[50] Yang IV, Fingerlin TE, Evans CM, Schwarz MI, Schwartz DA (2015). MUC5B and idiopathic pulmonary fibrosis. Ann. Am. Thorac. Soc..

[51] Zou J, Li Y, Yu J, Dong L, Husain AN, Shen L (2020). Idiopathic pulmonary fibrosis is associated with tight junction protein alterations. Biochim. Biophys. Acta Biomembr..

[52] Paw M, Wnuk D, Jakieła B, Bochenek G, Sładek K, Madeja Z (2021). Responsiveness of human bronchial fibroblasts and epithelial cells from asthmatic and non-asthmatic donors to the transforming growth factor-β1 in epithelial-mesenchymal trophic unit model. BMC Mol. Cell Biol..

[53] Abs V, Bonicelli J, Kacza J, Zizzadoro C, Abraham G. Equine bronchial fibroblasts enhance proliferation and differentiation of primary equine bronchial epithelial cells co-cultured under air-liquid interface. PLoS One. 2019;14(11). 10.1371/journal.pone.0225025 PMC6853605 31721813

[54] Wyss Balmer T, Ansó J, Muntane E, Gavaghan K, Weber S, Stahel A (2017). In-vivo electrical impedance measurement in mastoid bone. Ann. Biomed. Eng..

[55] Cho MH, McDonald MLN, Zhou X, Mattheisen M, Castaldi PJ, Hersh CP (2014). Risk loci for chronic obstructive pulmonary disease: A genome-wide association study and meta-analysis. Lancet Respir. Med..

[56] Sun T, Huang Z, Liang WC, Yin J, Lin WY, Wu J, et al. TGFβ2 and TGFβ3 isoforms drive fibrotic disease pathogenesis. Sci. Transl. Med. 2021;13(605). 10.1126/scitranslmed.abe0407 34349032

[57] Asghar S, Monkley S, Smith DJF, Hewitt RJ, Grime K, Murray LA (2023). Epithelial senescence in idiopathic pulmonary fibrosis is propagated by small extracellular vesicles. Respir. Res..

[58] Laucho-Contreras ME, Polverino F, Gupta K, Taylor KL, Kelly E, Pinto-Plata V (2015). Protective role for club cell secretory protein-16 (CC16) in the development of COPD. Eur. Respir. J..

[59] Yanai H, Shteinberg A, Porat Z, Budovsky A, Braiman A, Zeische R (2015). Cellular senescence-like features of lung fibroblasts derived from idiopathic pulmonary fibrosis patients. Aging (Albany NY)..

[60] Lin Y, Xu Z (2020). Fibroblast senescence in idiopathic pulmonary fibrosis. Front. Cell Dev. Biol..

[61] Ma H, Wu X, Li Y, Xia Y (2022). Research progress in the molecular mechanisms, therapeutic targets, and drug development of idiopathic pulmonary fibrosis. Front. Pharmacol..

[62] Pitsalidis C, Van Niekerk D, Moysidou CM, Boys AJ, Withers A, Vallet R (2022). Organic electronic transmembrane device for hosting and monitoring 3D cell cultures. Sci. Adv..

