# Supplementary material for: Modelling bronchial epithelial-fibroblast cross-talk in idiopathic pulmonary fibrosis (IPF) using a human-derived in vitro air liquid interface (ALI) culture
Source: Sci Rep. 2024 Jan 2;14:240. doi: 10.1038/s41598-023-50618-y (PMC10761879; doi:10.1038/s41598-023-50618-y)
Supplement: Supplementary file 1 — Supplementary Information. [file 41598_2023_50618_MOESM1_ESM.docx]

**Additional file 1 – Supplementary figures**

**Modelling bronchial epithelial-fibroblast cross-talk in Idiopathic Pulmonary Fibrosis (IPF) using a human-derived in vitro Air Liquid Interface (ALI) culture.**

Sarah L. Barron^1^*, Owen Wyatt^2^, Andy O’Connor^2^, David Mansfield^3^, E. Suzanne Cohen^2^, Tomasz Witkos^4^, , Sam Strickson^2^ and Róisín M. Owens^1^*

## **
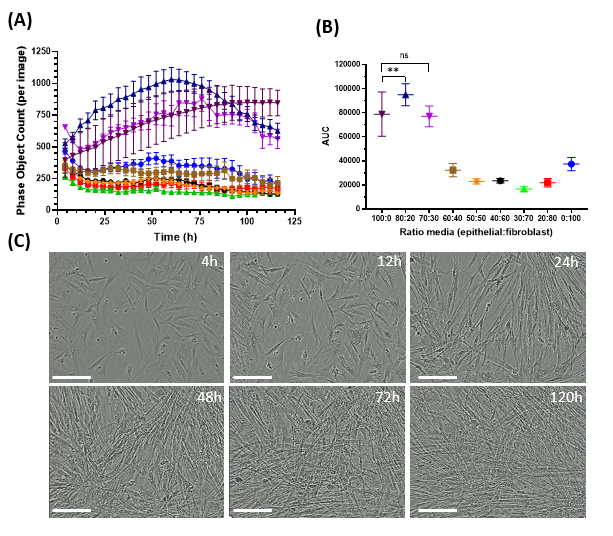
**

**Figure S1.** Co-culture media optimisations. When grown in different ratios of NHBE:HLF media, NHBE proliferation (A), and area under the curve (AUC) analysis (B), showed ratios below 70:30 negatively affected proliferation (** P = 0.0012, NS = 0.99). Fibroblast proliferation was negatively affected when grown in 100:0 media (data not shown), so 80:20 media was chosen as optimal. Brightfield images of fibroblasts grown in 80:20 media, over a period of 5 days, demonstrating no adverse effects on proliferation (C). n =12 and presented as the mean ± SD. Scale bars = 200 µm. NHBE = Normal Human Bronchial Epithelial cell; HLF = Human Lung Fibroblast.

**
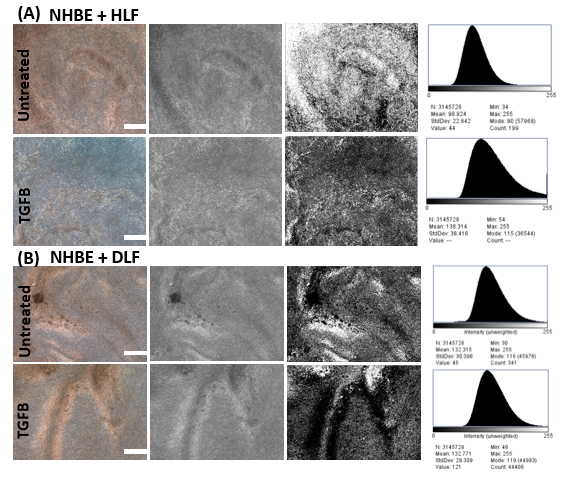
**

**Figure S2.** Representative brightfield image segmentations of apical muco-ciliary surface architecture of diseased (A) and healthy (B) co-cultures when untreated (top rows) or treated (bottom row). Brightfield (left column), greyscale (middle left column), contrast (middle right) and greyscale histogram plots (right column) for surface contrast quantification. Scale bars = 1mm. . NHBE = Normal Human Bronchial Epithelial cell; NHLF = Normal Human Lung Fibroblast; DHLF = Diseased Human Lung Fibroblasts.

**
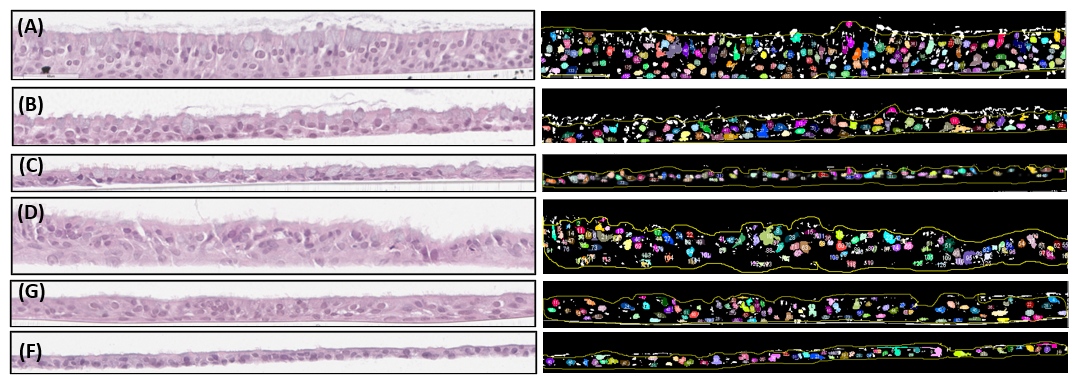
**

**Figure S3.** Representative histology sections and corresponding image segmentation analysis that were used to quantify nucleus morphology and cell density in untreated (A-C) and TGFβ treated (D-F) cultures. First and fourth rows = NHBE monocultures; Second and fifth rows = healthy co-cultures; Third and sixth rows = diseased co-cultures. Scale bars = 60 µm.

**
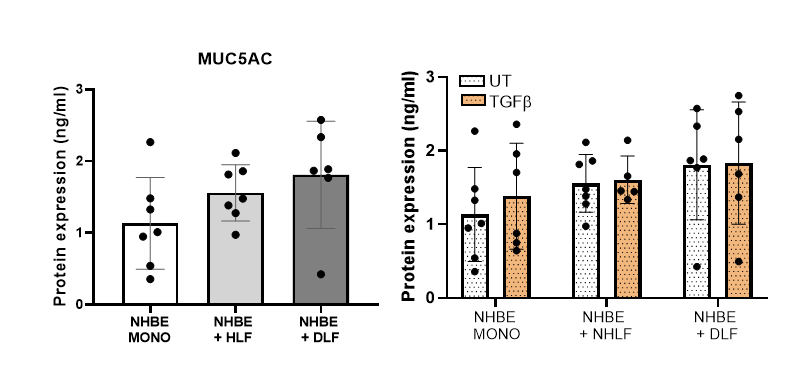
**

**Figure S4.** Comparison of MUC5AC protein expression in untreated or treated in vitro cultures. Comparisons of untreated cultures (left) were performed using a one-way ANOVA. For comparisons of untreated vs treated (right) multiple T tests per row with Holm-Sidak multiple comparison, and revealed no significance between groups or with treatment. n = 2 donors for NHBEs, n = 3 donors for NHLFs and n = 3 donors for DHLFs, N = 7-8 for each condition and presented as the mean ± SD.

**
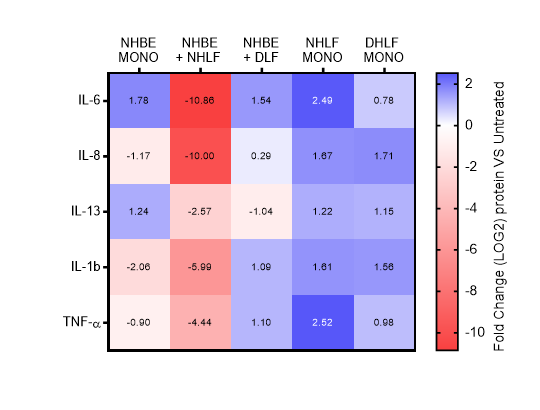
**

**Figure S5.** Change in cytokine release of in vitro cultures stimulated with TGFβ-1. Data are normalised and presented as fold log change to respective untreated groups. n = 2 donors for NHBEs, n = 3 donors for NHLFs and n = 3 donors for DHLFs, N = 4-8 for each condition and presented as the mean ± SD. NHBE = Normal Human Bronchial Epithelial cell; NHLF = Normal Human Lung Fibroblast; DHLF = Diseased Human Lung Fibroblasts ; UT = untreated.


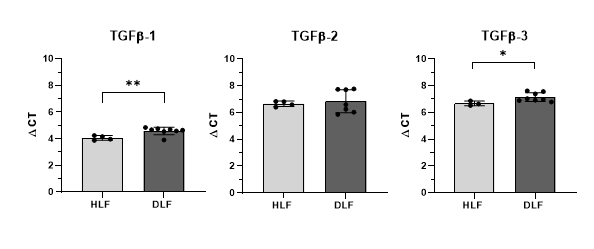


**Figure S6.** Comparison of TGFβ isoforms in untreated healthy vs diseased fibroblasts. (**, P = 0.0083;*, P = 0.045) in untreated DHLFs vs NHLFS. Comparisons were performed using an unpaired T test. ΔCT = CT_(GAPDH)_ – CT_(Gene of Interest)_. n = 2 donors for NHBEs, n = 3 donors for NHLFs and n = 3 donors for DHLFs, N = 4-8 for each condition and presented as the mean ± SD.


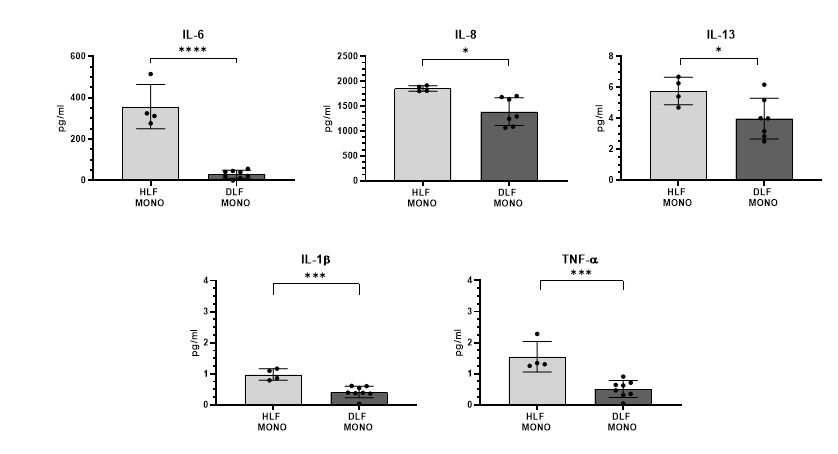


**Figure S7.** Comparison of cytokine release in response to TGFβ-1 stimulation in healthy vs diseased fibroblasts. Comparisons were performed using an unpaired T test. (****, P = <0.0001; *, P = 0.0105; *, P = 0.041; ***, P = 0.005; ***, P = 0.007). n = 2 donors for NHBEs, n = 3 donors for NHLFs and n = 3 donors for DHLFs, N = 4-8 for each condition and presented as the mean ± SD.
